# The Significance of Trust in Washington State's Aging Network COVID Response

**DOI:** 10.1093/geroni/igab046.020

**Published:** 2021-12-17

**Authors:** Clara Berridge, Ian Johnson, Callie Freitag, Carolyn Parsey, Maggie Ramirez

**Affiliations:** 1 University of Washington, Seattle, Washington, United States; 2 University of Washington, SEATTLE, Washington, United States; 3 University of Washington School of Public Health, Seattle, Washington, United States

## Abstract

In late summer of 2020, we interviewed 45 senior leaders of social services and health care organizations serving older adults throughout Washington State about service demand, new challenges, and organizational adaptations. These organizations work with people made particularly vulnerable in the pandemic. A significant share reported that half or more of their clients live at or below the poverty line (54%), are people of color (29%), or have limited English language proficiency (20%). The state’s aging network leveraged strong partnerships, expertise, and community knowledge to provide trusted essential services to older Washingtonians and their caregivers. The role of trust as an enabler of emergency response and connection in the context of gentrification, the digital divide, employment loss, and language service gaps will be discussed, as will lack of trust as a barrier to service access, particularly for Latinx immigrant and migrant older adults.

